# Supplementary material for: High-efficiency RNA-based reprogramming of human primary fibroblasts
Source: Nat Commun. 2018 Feb 21;9:745. doi: 10.1038/s41467-018-03190-3 (PMC5821705; doi:10.1038/s41467-018-03190-3)
Supplement: Supplementary file 1 — Supplementary Information [file 41467_2018_3190_MOESM1_ESM.pdf]

Supplementary Figure 1

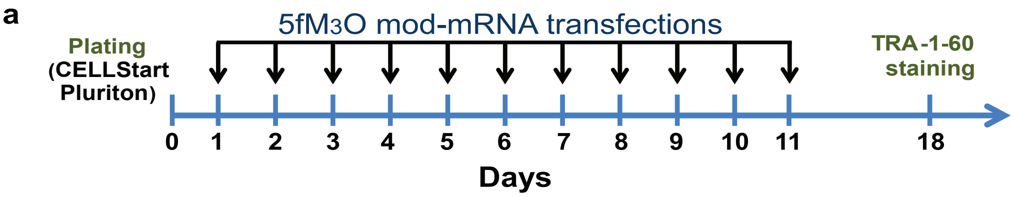

b

Cells plated/well (6-well format)	Neonatal lines	Number of TRA-1-60 positive colonies/well	Efficiency (%): Number of TRA-1-60 positive colonies/number of input cells x 100%
**100,000**	FN1	0	0
	FN2	0	0
**50,000**	FN1	216.3 ± 50.96	0.43 ± 0.1
	FN2	40.3 ± 11.59	0.081 ± 0.023
**10,000**	FN1	0	0
	FN2	0	0

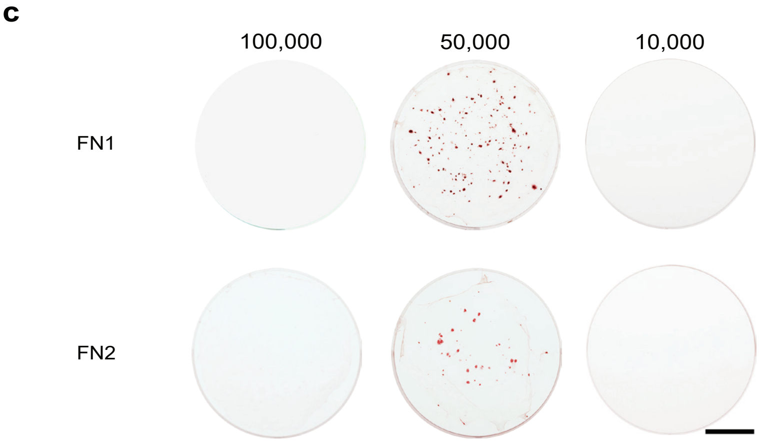

Supplementary Figure 1: Generation of human iPSCs with a previously reported M_3O-based mod-mRNA reprogramming approach.

(a) Schematic diagram of a $5fM_3O$ mod-mRNA transfection regimen during feeder-free reprogramming as previously described[1].
(b) Summary table showing the yield of TRA-1-60 positive colonies and the resulting reprogramming efficiency at day 18 of the reprogramming regimen depicted in (a), initiated at 3 different densities (100,000 cells/well, 50,000 cells/well, or 10,000 cells/well of a 6-well format dish), using two independent primary neonatal fibroblast lines (FN1 and FN2). Mean ± s.d. (n = 3).
(c) Representative TRA-1-60-stained reprogramming wells corresponding to conditions in (b) with the indicated number of input cells. Note that the initial seeding density of input cells needed to be pre-determined empirically for this protocol. The working seeding density appeared to be 50,000 input cells/well of a 6-well format dish for two independent human primary neonatal fibroblast lines. The higher (100,000 cells/well) and lower (10,000 cells/well) plating densities did not produce any TRA-1-60 positive colonies either due to cell overcrowding in higher density plating, or cell death in lower density plating. Scale bar, 10 mm.

Supplementary Figure 2

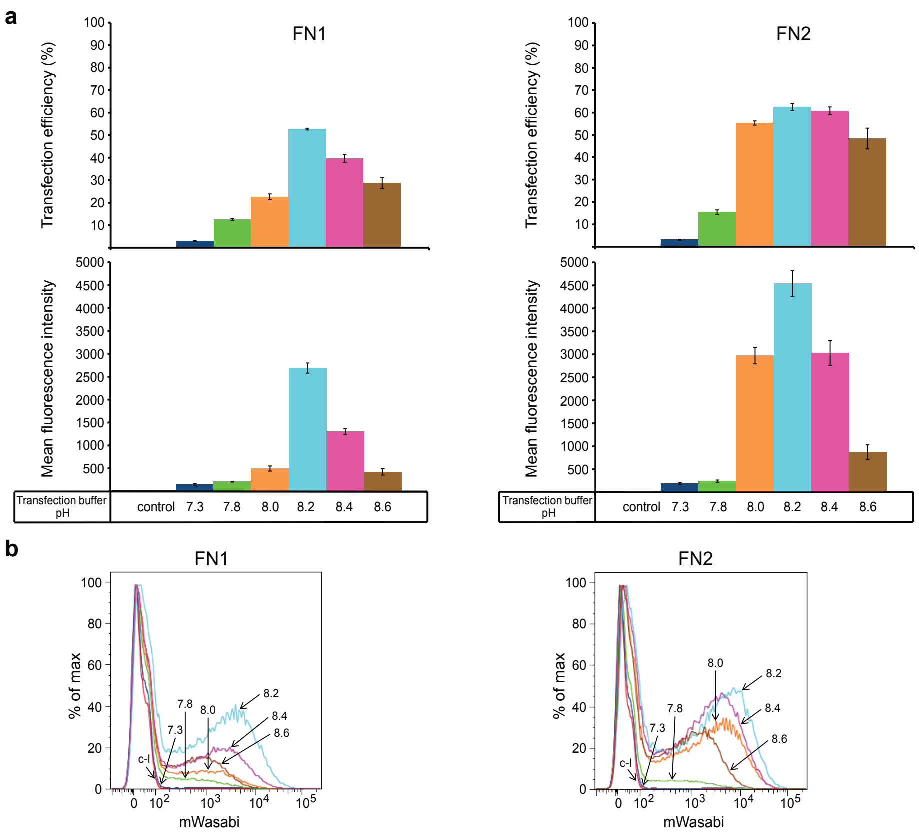

Supplementary Figure 2: Optimizing transfection efficiency of mWasabi mod-mRNA in human primary fibroblasts.

(a) Transfection efficiency (top) and mean fluorescence intensity (bottom) of human primary neonatal fibroblasts (FN1, left, and FN2, right) transfected with 500 ng of mod-mRNA encoding mWasabi, using Opti-MEM as the transfection buffer and KOSR as a plating medium, as determined by flow cytometry 24 h post transfection. The pH of the transfection buffer (Opti-MEM) is indicated for each transfection condition. Error bars, mean ± s.d. for all panels ($n = 3$).
(b) Representative flow cytometry histograms of mWasabi expression corresponding to conditions in (a) for FN1 and FN2. The pH of the transfection buffer (Opti-MEM) is indicated for each histogram. Histogram colors correspond to bar colors in (a).

Supplementary Figure 3

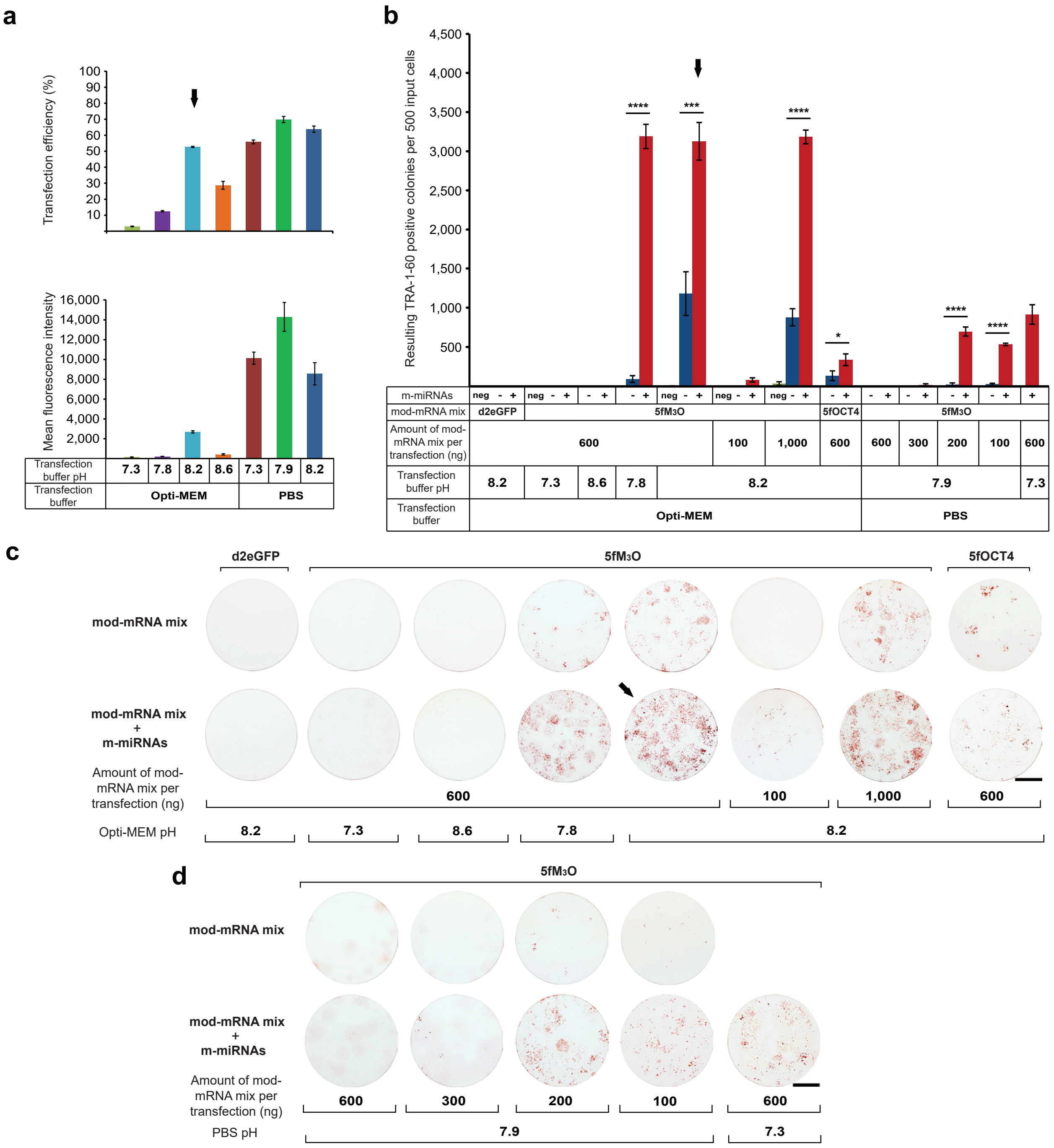

Supplementary Figure 3: Validating the reproducibility of the optimized RNA-based reprogramming regimen using an independent fibroblast line.

All conditions presented replicate Fig. 1 using an independent human primary neonatal fibroblast line, FN1.

(a) Transfection efficiency (top) and mean fluorescence intensity (bottom) of human primary neonatal fibroblasts (FN1) transfected with 500 ng of mod-mRNA encoding mWasabi using the indicated transfection buffers, as determined by flow cytometry 24 h post transfection. Error bars, mean ± s.d. for all panels (n = 3).

(b) Effect of mod-mRNA titration and the addition of m-miRNAs on the reprogramming of human primary neonatal fibroblasts (FN1). All reprogramming conditions were initiated with 500 cells/well of a 6-well format dish. Cells were transfected every 48 h with differing amounts of mod-mRNA encoding mWasabi (transfection control) and either d2eGFP as a negative control or 6-factor reprogramming cocktails containing either M_3O (5fM_3O) or OCT4 (5fOCT4). Mod-mRNA transfections were performed alone or in combination with reprogramming m-miRNAs or Neg. Control siRNA (neg) transfections, using the indicated transfection buffers. Numbers of resulting TRA-1-60 positive colonies on day 18 of the indicated regimens are plotted. Error bars, mean ± s.d. (n = 3). The yield of TRA-1-60 positive colonies was compared between the regimens performed in the presence or absence of m-miRNAs. *P* values were calculated using the unpaired two-tailed Student's t-test. *$P < 0.05$, ***$P < 0.001$, ****$P < 0.0001$.

(c) Representative TRA-1-60-stained reprogramming wells corresponding to conditions indicated in (b) for Opti-MEM as the transfection buffer. Scale bar, 10 mm.

(d) Representative TRA-1-60-stained reprogramming wells corresponding to conditions indicated in (b) for PBS as the transfection buffer. Scale bar, 10 mm.

Solid black arrows indicate optimal conditions for mod-mRNA transfections in (a) and iPSC colony generation in (b) and (c).

Supplementary Figure 4

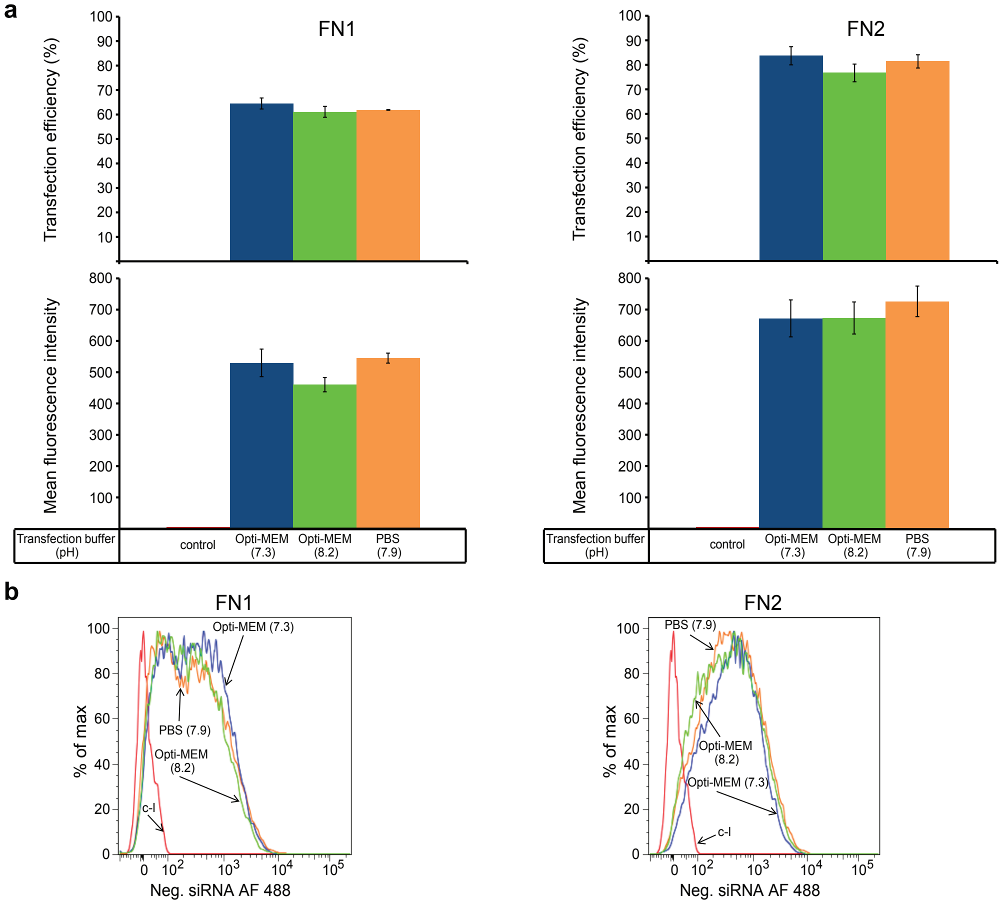

Supplementary Figure 4: Transfection efficiency of fluorescently labeled Neg. Control siRNA is independent of transfection buffer composition or pH.

(a) Transfection efficiency (top) and mean fluorescence intensity (bottom) of human primary neonatal fibroblasts (FN1, left and FN2, right) transfected with 20 pmoles of Alexa Fluor 488 conjugated AllStars Neg. siRNA (QIAGEN), using either Opti-MEM (pH 7.3 or pH 8.2) or PBS (pH 7.9) as transfection buffers and KOSR as a plating medium, as determined by flow cytometry 24 h post transfection. The pH of the transfection buffer (Opti-MEM or PBS) is indicated for each transfection condition. Error bars, mean ± s.d. for all panels ($n = 3$).
(b) Representative flow cytometry histograms of Neg. siRNA AF 488 fluorescence corresponding to conditions in (a) for FN1 and FN2. The pH of transfection buffers (Opti-MEM or PBS) is indicated for each histogram. Histogram colors correspond to bar colors in (a).

Supplementary Figure 5

Cells plated/well	TRA-1-60 positive colonies/well	Efficiency (%)
100,000	4.7 ± 5.51	0.0053 ± 0.0055
50,000	9.3 ± 12.7	0.0187 ± 0.0254
10,000	1,278.0 ± 575.32	12.78 ± 5.753
5,000	1,772.0 ± 576.6	35.44 ± 11.53
1,000	**2,928.7** ± 45.32	**292.87** ± 4.53
500	**3,132.0** ± 240.04	**626.40** ± 48.01
200	**1,396.3** ± 493.66	**698.17** ± 246.83

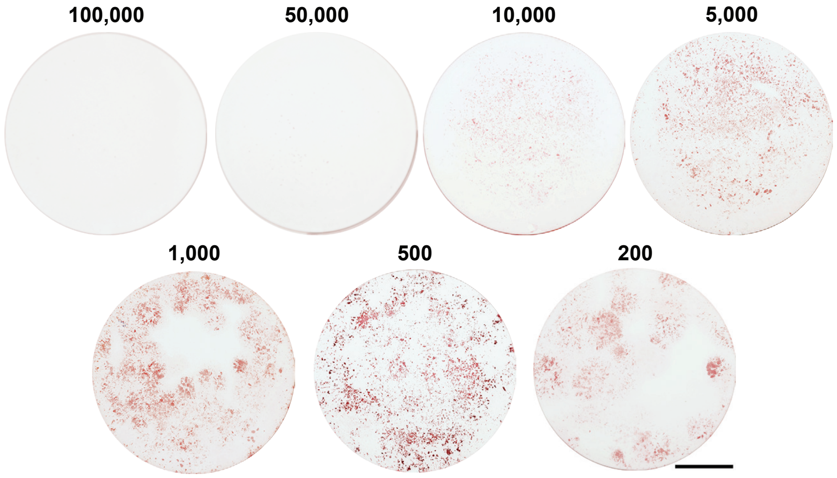

Supplementary Figure 5: Low initial plating densities enhance reprogramming of an independent primary neonatal fibroblast line.

Summary table and representative TRA-1-60-stained reprogramming wells show the yield of TRA-1-60 positive colonies at day 18 of the optimized RNA-based reprogramming regimen (Fig. 1b), initiated with human primary neonatal fibroblasts (FN1) at the indicated plating densities in a 6-well format dish. Mean ± s.d. (n = 3). Scale bar, 10 mm.

Supplementary Figure 6

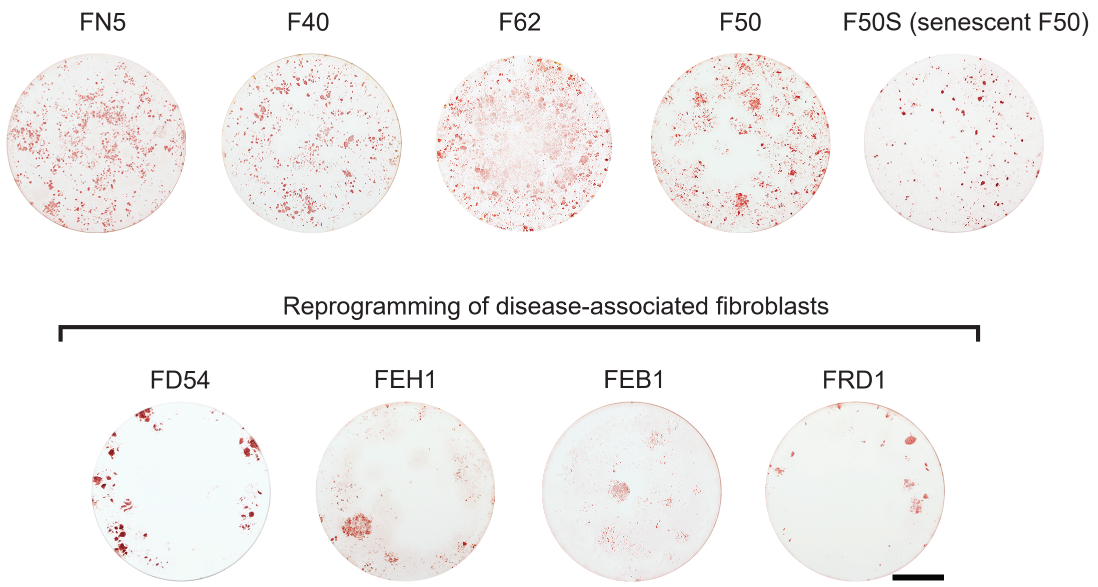

Supplementary Figure 6: Reprogramming of a variety of human fibroblast lines with the optimized RNA-based approach.

Representative TRA-1-60-stained reprogramming wells correspond to reprogramming conditions in Table 1 and show the yield of TRA-1-60 positive colonies at day 18 of the optimized RNA-based reprogramming regimen (Fig. 1b), initiated at plating densities indicated in Table 1. The representative wells are presented for the reprogramming of a primary neonatal fibroblast line (FN5), three healthy primary adult fibroblast lines (F40, F62, and F50), a senescent fibroblast line (F50S), a fibroblast line from an individual with Down Syndrome (FD54), and three lines derived from patients with inherited skin blistering diseases: epidermolytic ichthyosis (FEH1), epidermolysis bullosa simplex (FEB1), and recessive dystrophic epidermolysis bullosa (FRD1).

Supplementary Figure 7

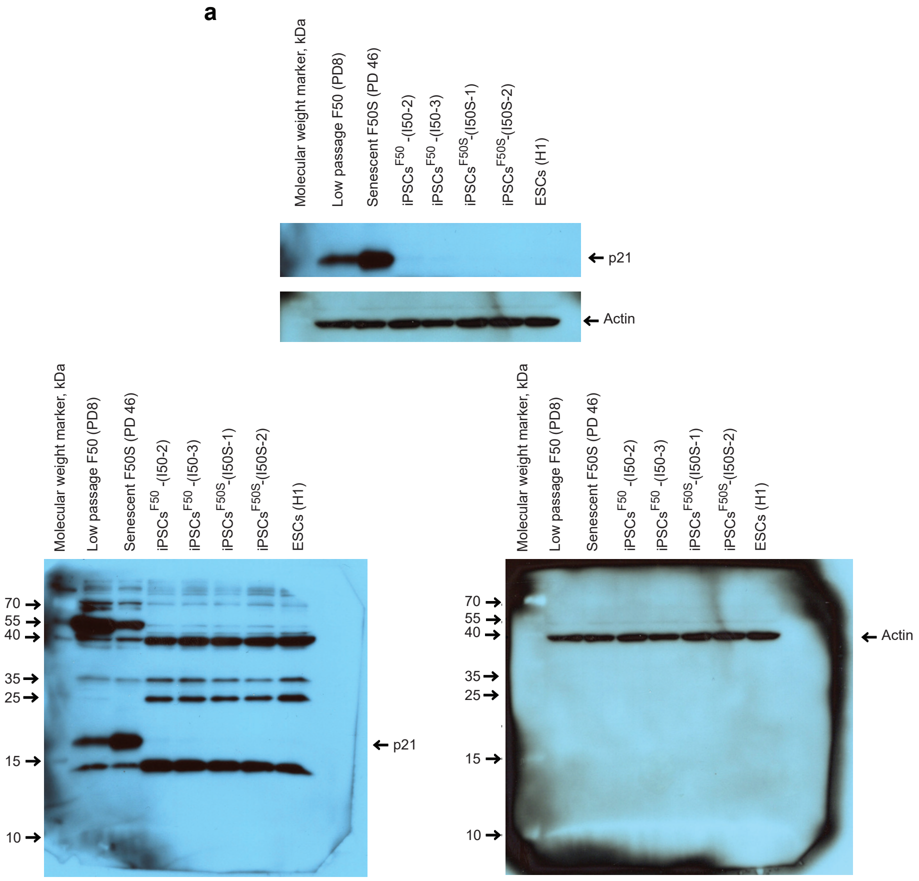

Supplementary Figure 7: The senescence-associated protein p21 is downregulated in iPSC lines.

(a) Western blot analysis showing the downregulation of the senescence-associated protein p21 in iPSC lines generated from low passage F50 (I50-2 and I50-3) and senescent F50S (I50S-1 and I50S-2) fibroblasts. Human ESCs (H1) were used as a control. See Supplementary Table 1 for iPSC clones derived from F50 and F50S.

(b) Uncropped scans of the western blots presented in (a) with the location of a molecular weight marker.

Supplementary Figure 8

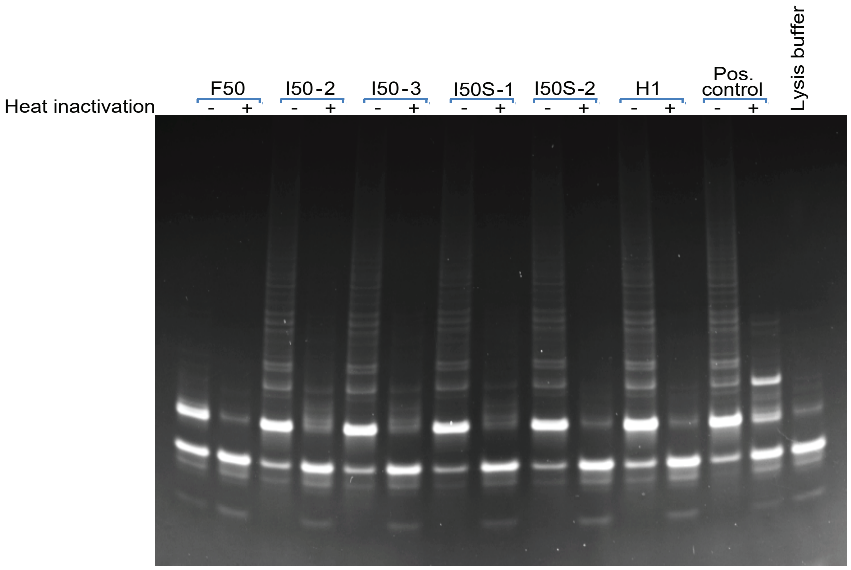

Supplementary Figure 8: Reactivation of telomerase in iPSC lines.

The analysis of telomerase activity in F50, in iPSCs lines derived from F50 and F50S, and in H1 using the Trapeze Telomerase Detection Kit (Chemicon). Note the ladder pattern of telomerase-elongated products in lanes corresponding to iPSC and ESC lines that is identical to the pattern of products in the positive control sample (pos. control). The ladder is absent in the fibroblast sample F50. Heat inactivation inhibits telomerase activity, resulting in the disappearance of the ladder of products in telomerase-positive samples. The lysis buffer and heat inactivation of samples are used to assess the background of the assay. The telomerase activity is high in all iPSCs and results in the elongation of telomeres in live cells (Fig. 5b).

Supplementary Figure 9

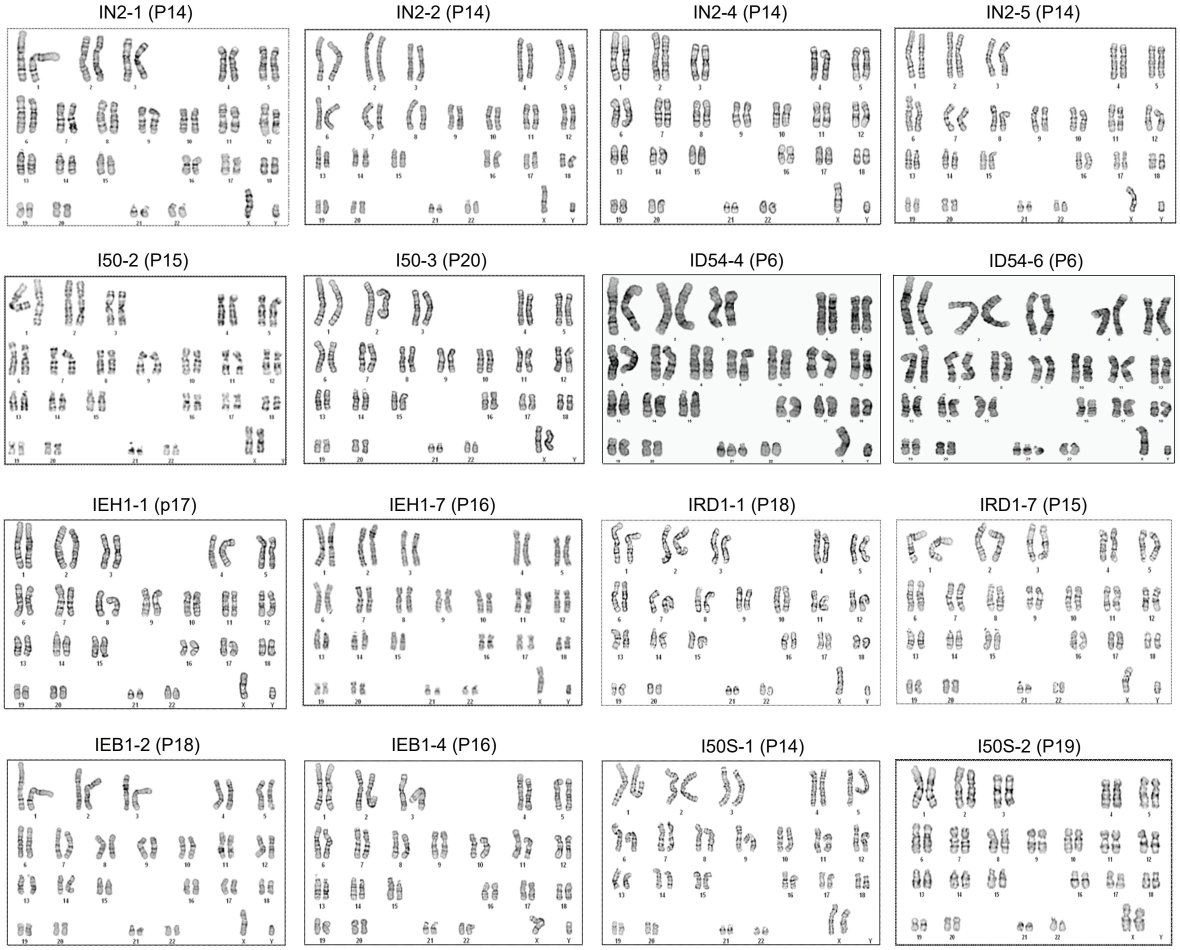

Supplementary Figure 9: Karyotyping of selected iPSC lines.

Representative karyotype images show an expected chromosomal composition for each of the indicated iPSC lines. See Supplementary Table 1 for the results of G-banded chromosome analysis and the lines that were subjected to karyotyping.

Supplementary Figure 10

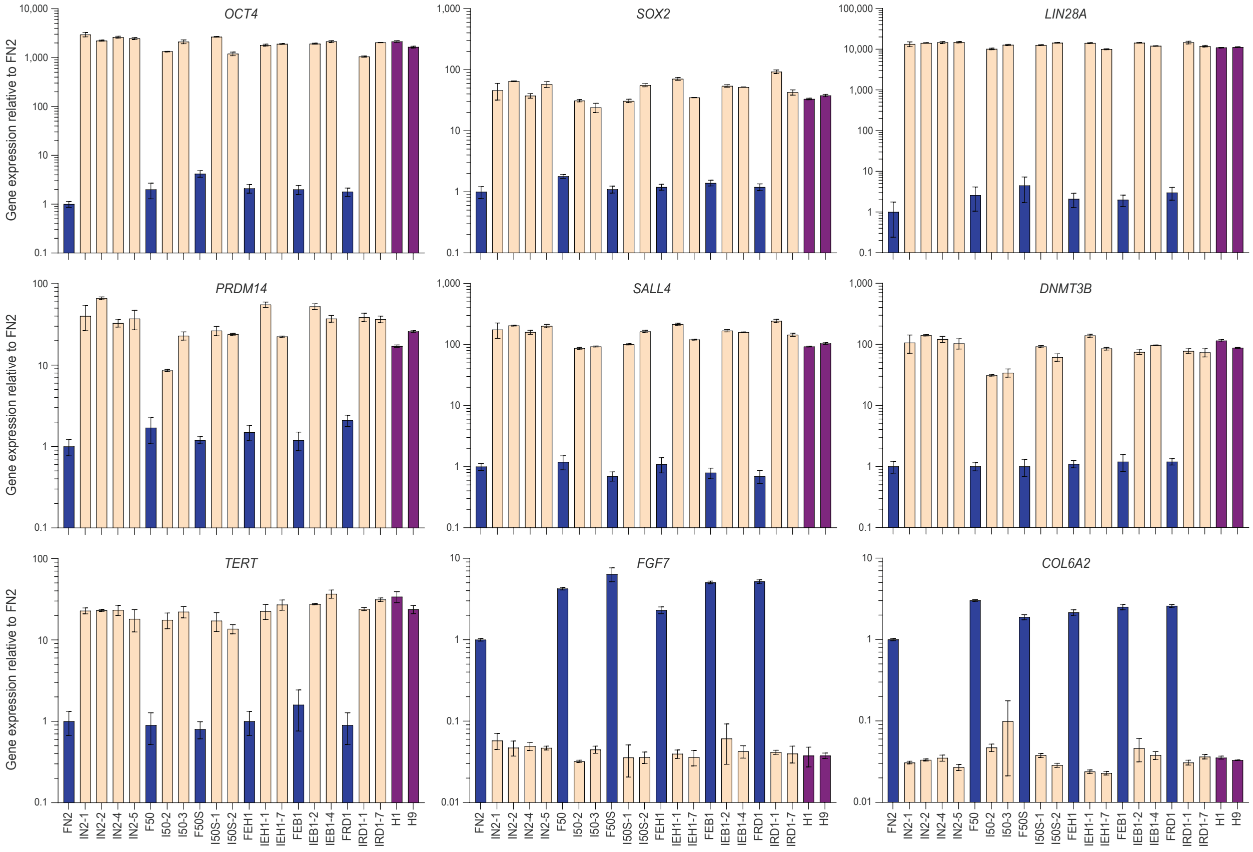

Supplementary Figure 10: The generated human iPSCs and human ESCs have similar gene expression profiles.

Purified total RNA was extracted from generated human iPSCs, parental fibroblasts, and ESCs, and the expression of selected genes was profiled with the Nanostring nCounter Gene Expression Assay. Raw mRNA counts for selected genes were normalized to mRNA counts obtained for reference genes (See Supplementary Data 1) and compared to mRNA counts for the FN2 fibroblast sample. Fold changes in gene expression relative to FN2 are presented for parental fibroblast lines (FN2, F50, F50S, FEH1, FEB1, FRD1, blue color bars), the corresponding generated iPSC lines (IN2-1, IN2-2, IN2-4, IN2-5, I50-2, I50-3, I50S-1, I50S-2, IEH1-1, IEH1-7, IEB1-2, IEB1-4, IRD1-1, IRD1-7, peach color bars), and control ESC lines (H1, H9, purple color bars). Bars are labeled on the X axis and fold-changes are indicated on the Y axis. Error bars, mean ± s.d. for all panels (n = 3). Each bar represents data from 3 biological repeats. The expression of genes that are normally upregulated in ESC lines (*OCT4*, *SOX2*, *LIN28A*, *PRDM14*, *SALL4*, *DNMT3B*, *TERT*) significantly increases in the generated iPSCs as compared to parental fibroblast lines and reaches levels similar to those observed in ESC lines. In contrast, expression levels of the genes that are highly expressed in fibroblasts (*FGF7* and *COL6A2*) significantly decrease in the generated iPSC lines and reach levels similar to those observed in ESC lines. The gene expression data confirm the results of the RNA-Seq analysis presented in Fig. 6a.

Supplementary Figure 11

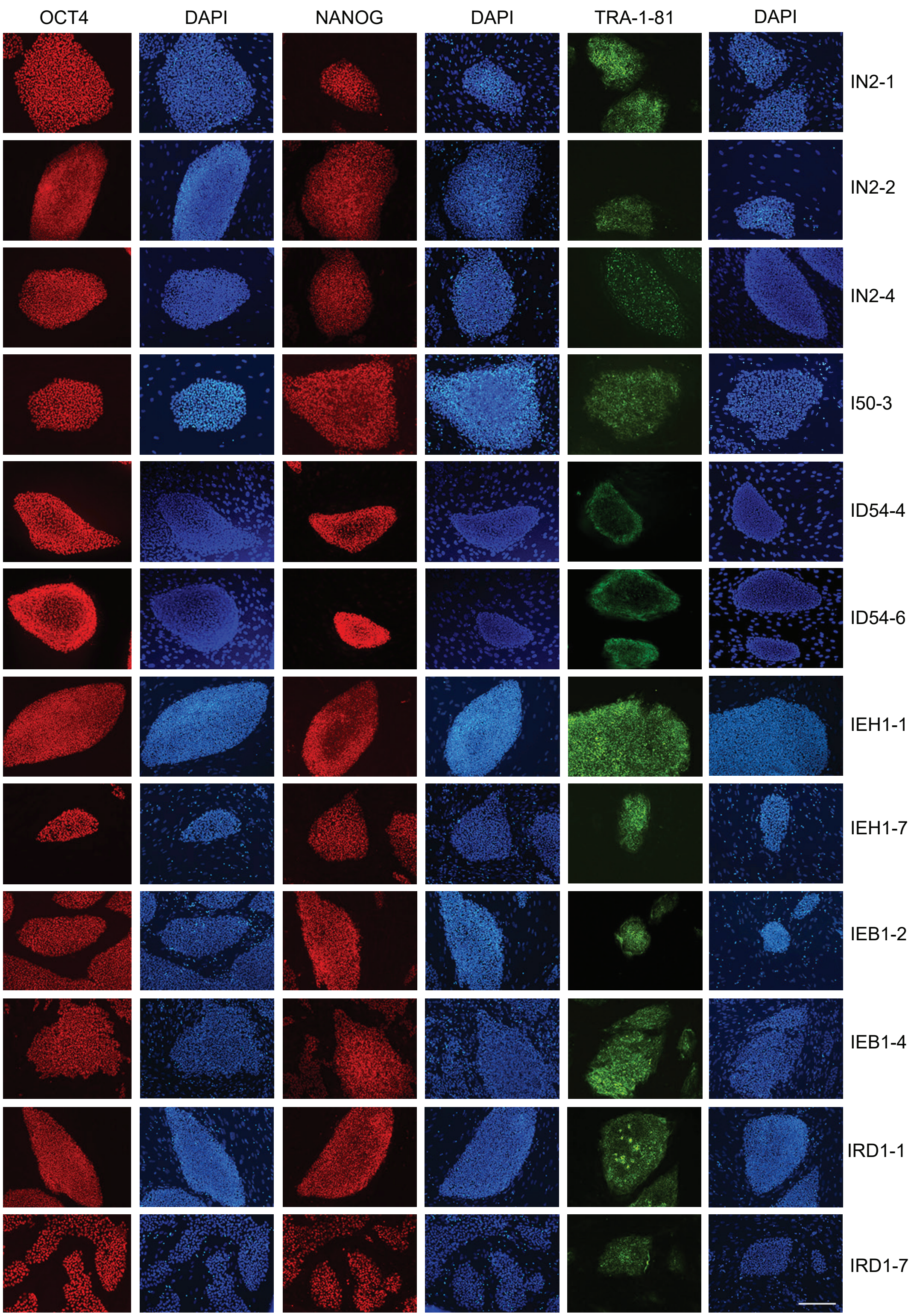

Supplementary Figure 11: Expression of pluripotency markers in selected iPSC lines.

Immunofluorescent analysis for the expression of OCT4, NANOG and TRA-1-81 was performed on the indicated iPSC lines. See Figs. 5c and 6b for additional iPSC lines, fibroblasts and ESC controls. Scale bar, 250 μm.

Supplementary Figure 12

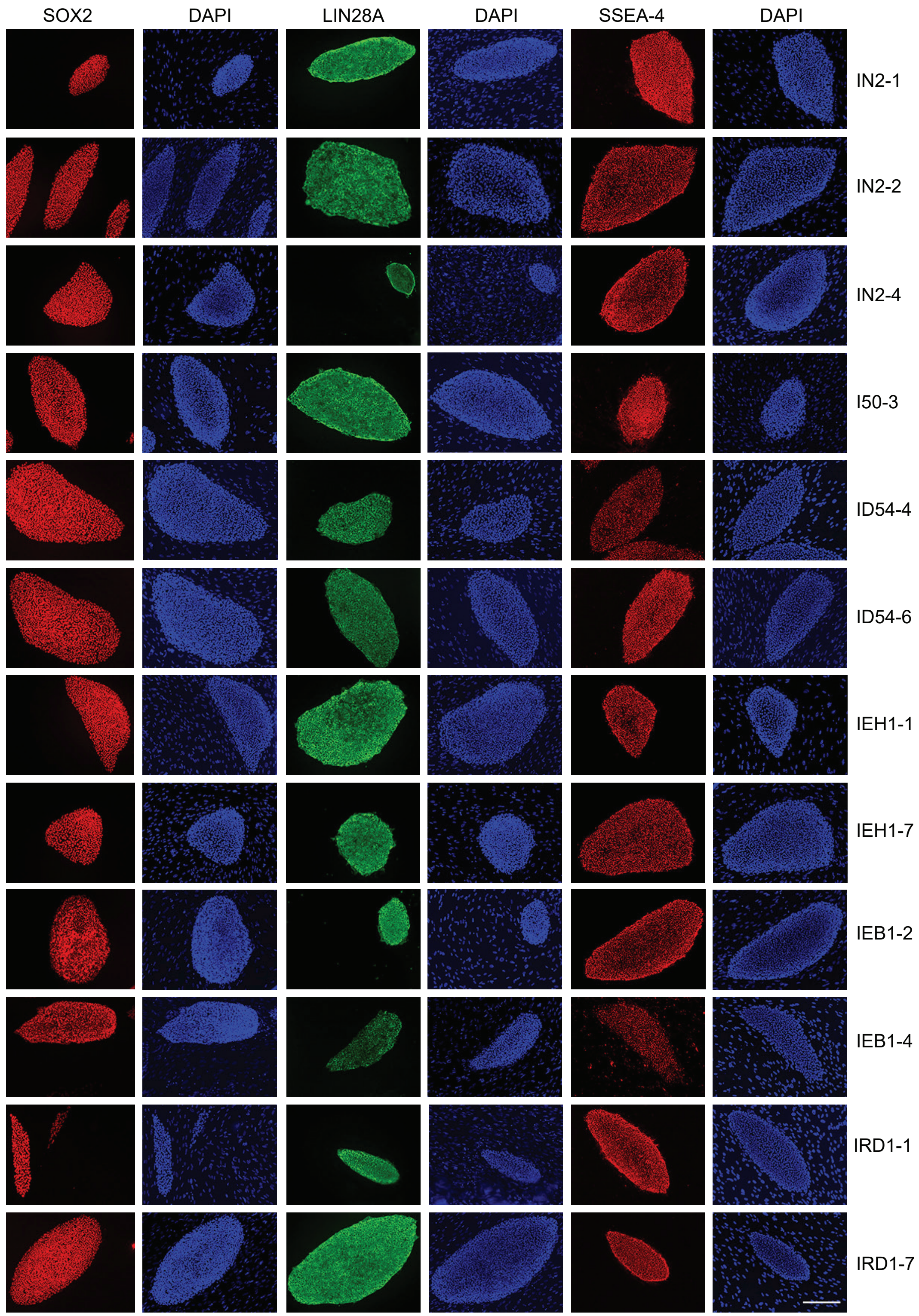

Supplementary Figure 12: Expression of additional pluripotency markers in selected iPSC lines.

Immunofluorescent analysis for the expression of SOX2, LIN28A and SSEA-4 was performed on the indicated iPSC lines. See Figs. 5c and 6b for additional iPSC lines, fibroblasts and ESC controls. Scale bar, 250 μm.

Supplementary Figure 13

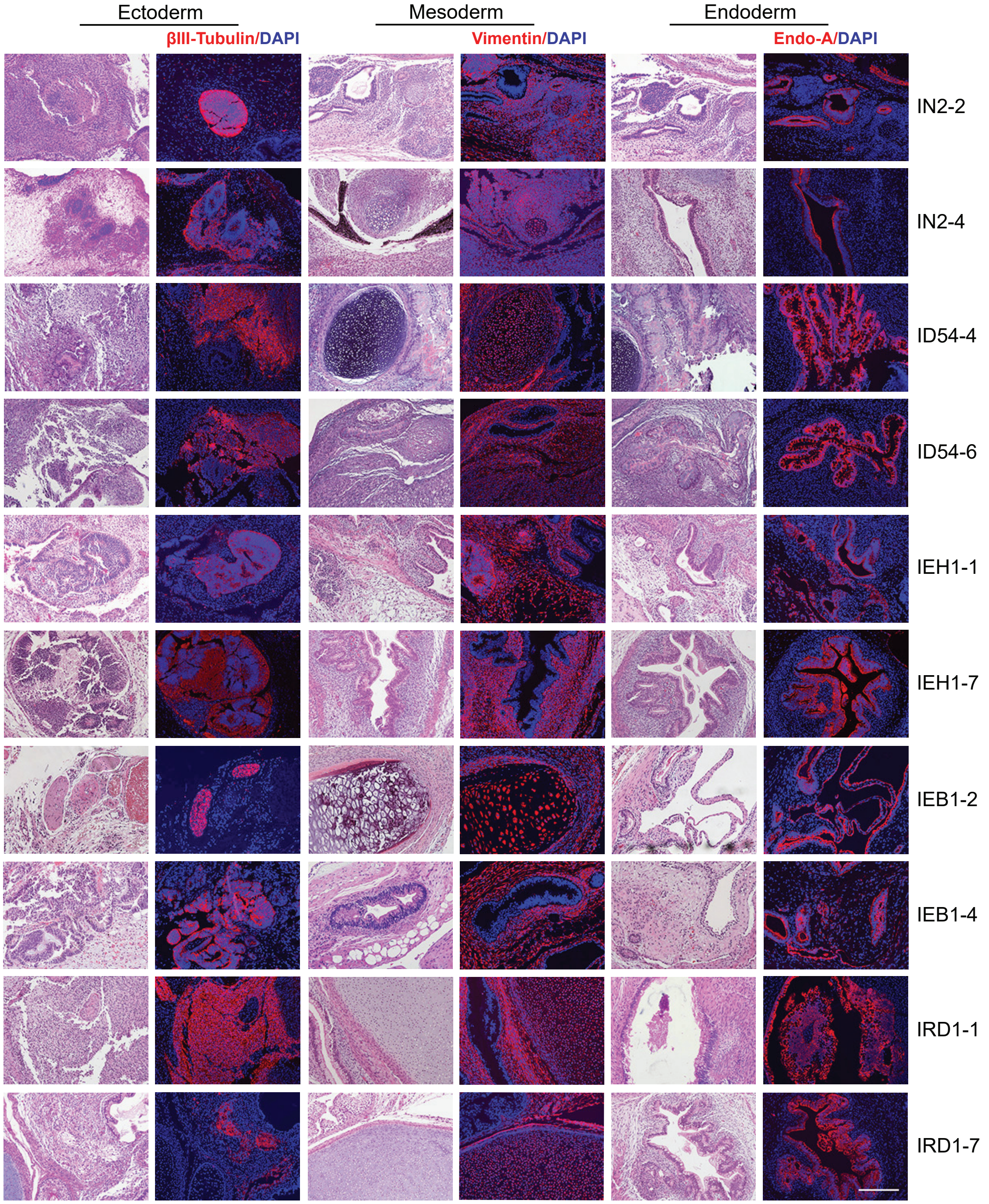

Supplementary Figure 13: Analysis of teratomas derived from selected iPSC lines.

Hematoxylin and eosin staining and immunofluorescent analysis of consecutive sections of teratomas derived from the indicated iPSC lines show histology and marker expression specific to ectoderm (TUJ1, neural tissues), mesoderm (vimentin, connective tissues), and endoderm (Endo-A, endothelium). See Figs. 5e and 6e for additional iPSC lines. Scale bar, 250 µm.

Supplementary Figure 14

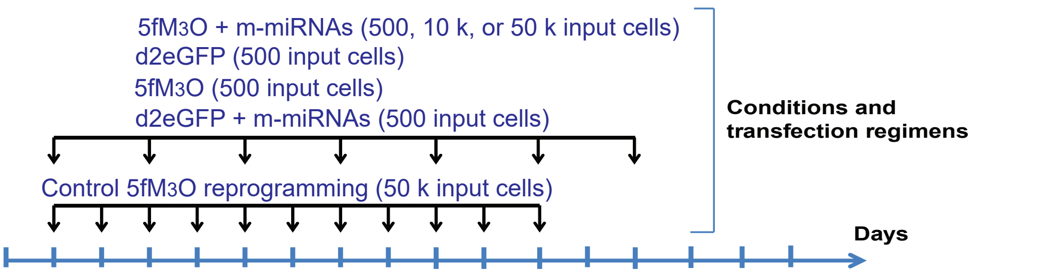

Sample collection

	1	2	3	4	5	6	7	8	9	10	11	12	13	14	15	16
Control 5fM3O reprogramming	X	X	X	X	X	X	X	X	X	X	X	X		X		X
5fM3O + m-miRNAs	X	X	X	X	X	X	X	X	X	X	X	X		X		X
d2eGFP		X		X		X		X		X		X		X		X
5fM3O		X	X	X	X	X	X	X	X	X		X		X		X
d2eGFP + m-miRNAs		X	X	X	X	X	X	X	X	X		X		X		X

Supplementary Figure 14: Schematic depicting the time course for analyzing gene expression in fibroblasts undergoing reprogramming.

Human primary neonatal fibroblasts were plated at the indicated densities per well of a 6-well format dish and subjected to reprogramming with either a control mod-mRNA protocol (control $5fM_3O$ reprogramming) as depicted in Supplementary Fig. 1a, or the optimized RNA-based reprogramming regimen as described in Fig. 1b ($5fM_3O$ + m-miRNAs). The optimized 48 h transfection regimen was used when reprogramming mod-mRNAs were delivered in the absence of m-miRNA transfections ($5fM_3O$), and when the delivery of a control mod-mRNA encoding d2eGFP was performed alone (d2eGFP) or in combination with reprogramming m-miRNAs (d2eGFP + m-miRNA). Control $5fM_3O$ reprogramming was performed in Pluriton medium with mod-mRNA delivered every 24 h. The rest of the conditions were performed in KOSR medium. The indicated time points (marked by X) were collected during each regimen to calculate population doubling and assess the activation of genes related to innate immunity, cell cycle and pluripotency using the Nanostring nCounter Gene Expression Assay. If a sample collection fell on the day of transfection, the samples were collected before transfection. The experiment was performed using two independent primary neonatal fibroblast lines (FN1 and FN2).

Supplementary Figure 15

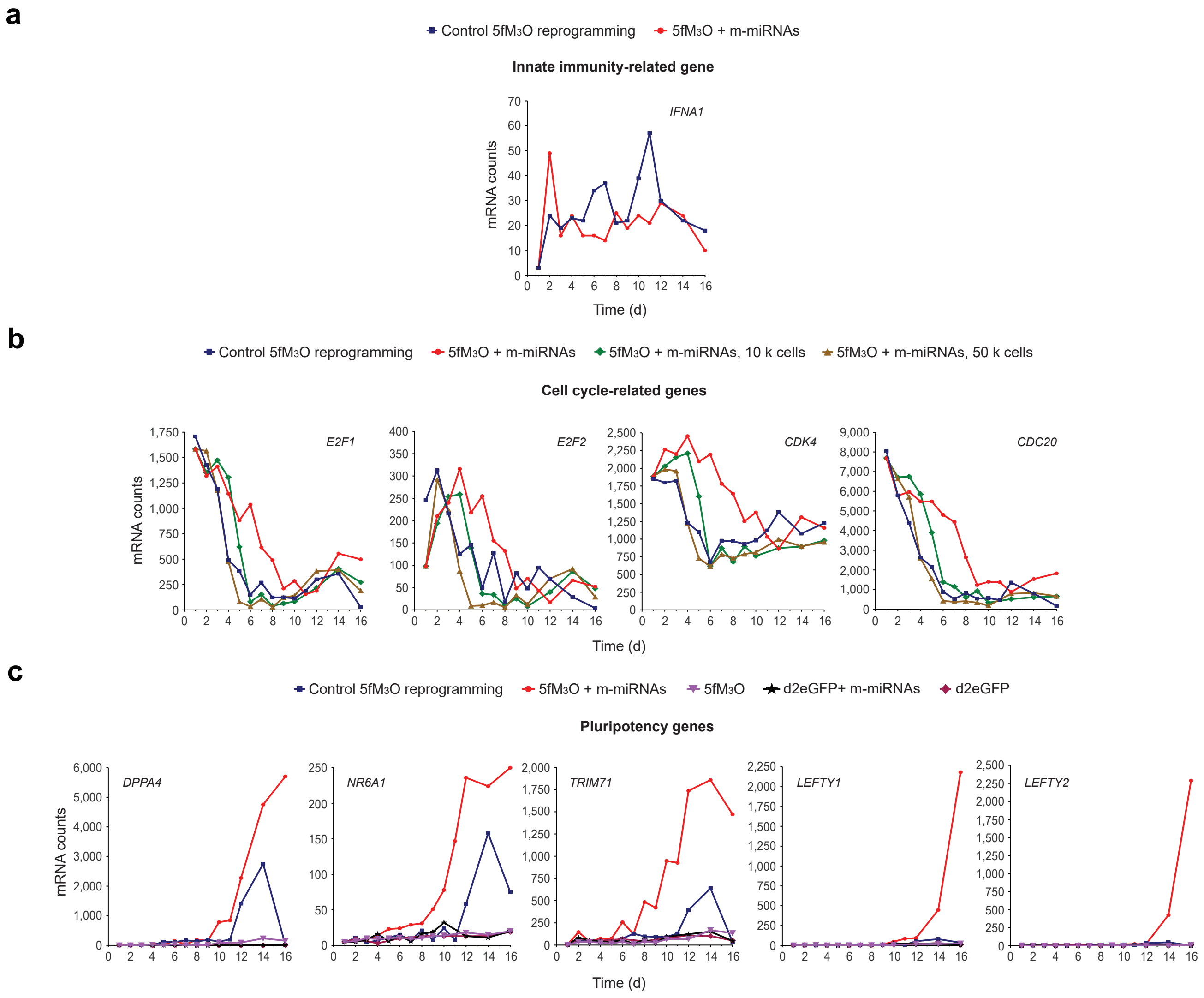

Supplementary Figure 15: A panel of additional genes analyzed in the time course experiment.

Normalized mRNA counts for additional genes were captured throughout the regimens performed with FN2 (as described in Fig. 7 and Supplementary Fig. 14).
(a) Graph showing normalized mRNA counts for the innate immunity-related gene *IFNA1* throughout reprogramming regimens.
(b) Graphs showing normalized mRNA counts for the indicated cell cycle-associated genes throughout the reprogramming regimens.
(c) Graphs showing normalized mRNA counts for selected pluripotency genes throughout the indicated regimens. The X axis shows time points (days) at which the samples were collected for analysis during the reprogramming regimens. The Y axis indicates the values for normalized mRNA counts.

Supplementary Figure 16

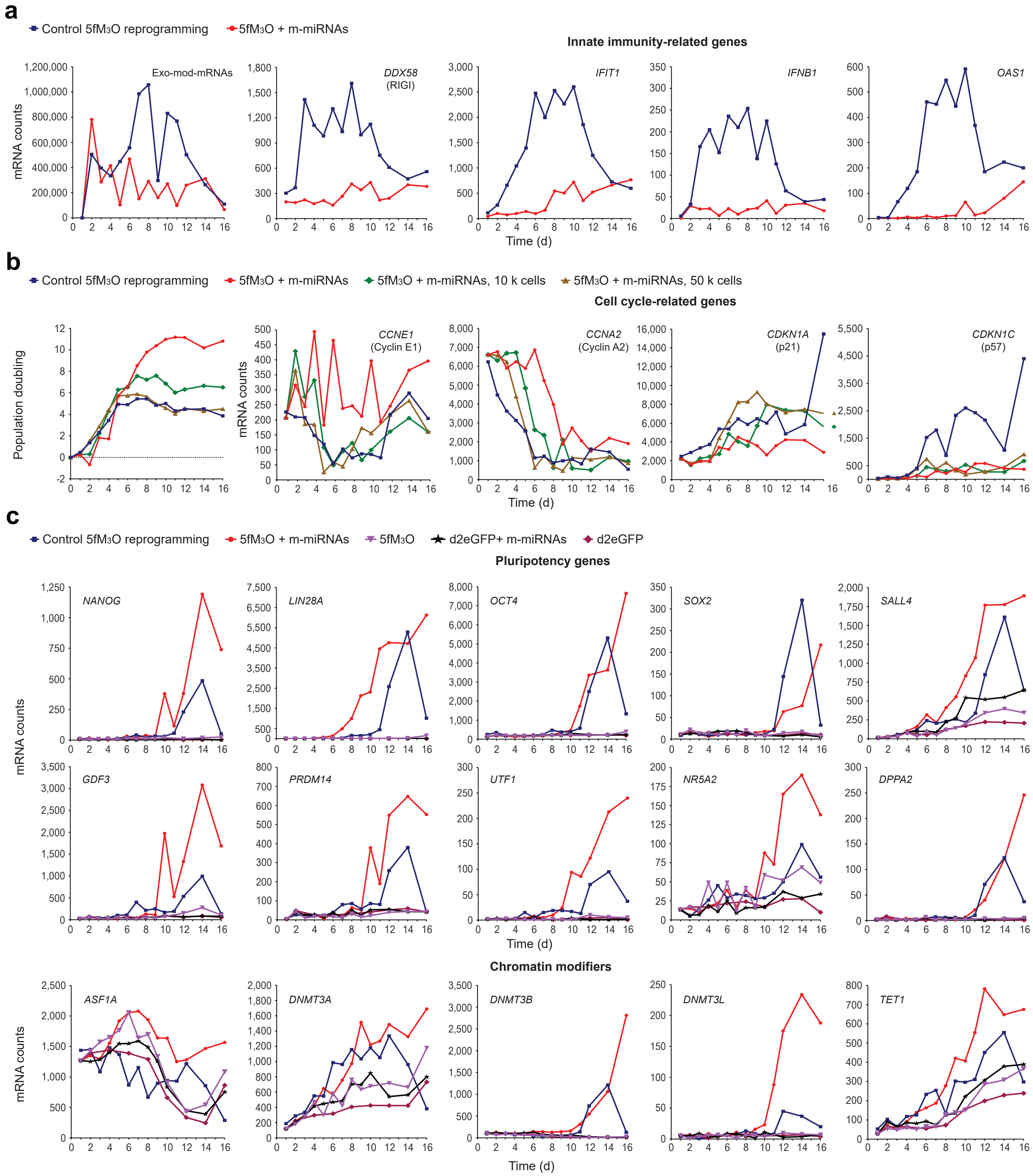

Supplementary Figure 16: Reduced innate immunity response and increased cell expansion enhance reprogramming of an independent fibroblast line.

The experimental conditions and analyses depicted in Fig. 7 and Supplementary Fig. 14 were recapitulated on an independent human primary neonatal fibroblast line (FN1).
(a) Graphs showing normalized mRNA counts for exogenous mod-mRNAs (Exo-mod-mRNAs) and innate immunity-related genes throughout the indicated reprogramming regimens as detected by the Nanostring nCounter Gene Expression Assay.
(b) Graphs summarizing population doubling (PD) and normalized mRNA counts for the indicated cell cycle-associated genes throughout the reprogramming regimens.
(c) Graphs showing normalized mRNA counts for selected pluripotency genes and chromatin modifier genes throughout the indicated regimens.
The X axis shows time points (days) at which the samples were collected for analysis during reprogramming regimens. The Y axis indicates values of either normalized mRNA counts or PD as specified on the corresponding plots. The analysis of additional genes associated with innate immunity, cell cycle, and pluripotency is shown in Supplementary Fig. 17.

Supplementary Figure 17

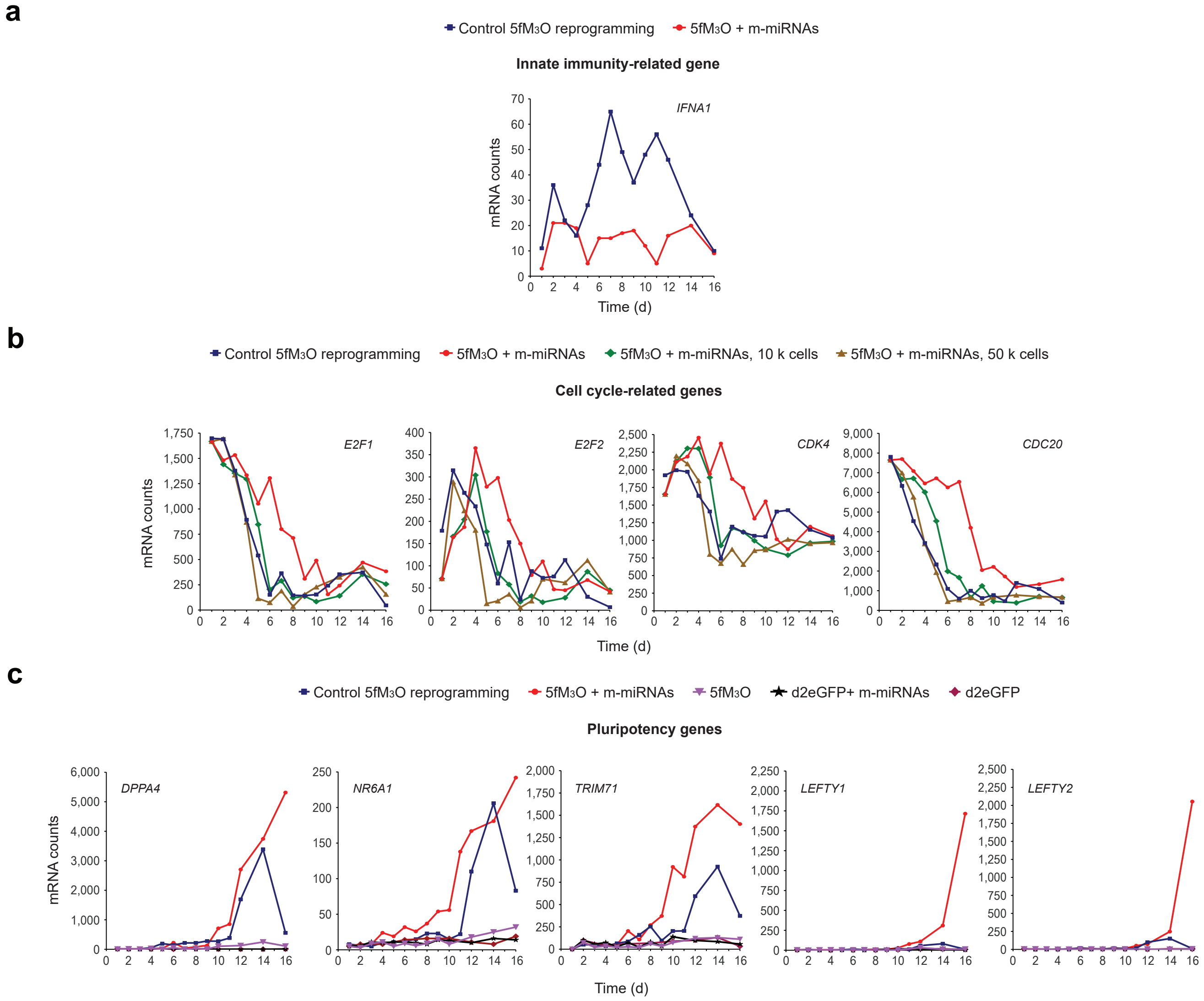

Supplementary Figure 17: A panel of additional genes analyzed in the time course experiment using an independent fibroblast line.

Normalized mRNA counts for additional genes were captured throughout the regimens performed with FN1 (as described in Supplementary Figs. 14 and 16).

(a) Graph summarizing normalized mRNA counts for the innate immunity-related gene *IFNA1* throughout the reprogramming regimens.

(b) Graphs showing normalized mRNA counts for the indicated cell cycle-associated genes throughout the reprogramming regimens.

(c) Graphs showing normalized mRNA counts for selected pluripotency genes throughout the indicated regimens.

The X axis shows time points (days) at which the samples were collected for analysis during the reprogramming regimens. The Y axis indicates values of normalized mRNA counts.

Supplementary Figure 18

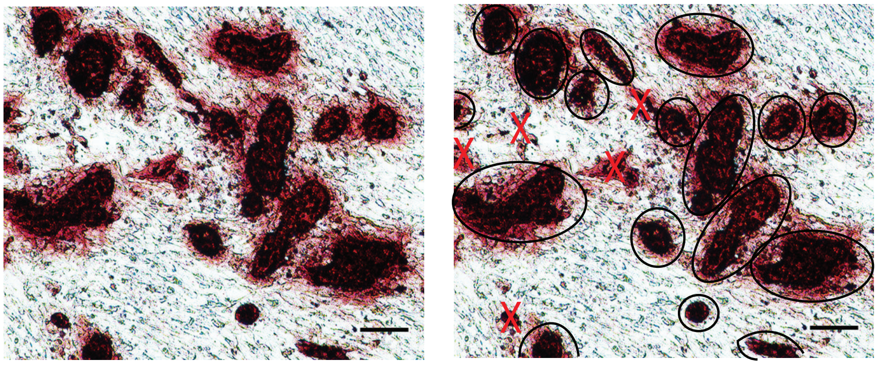

Supplementary Figure 18: Representative images of TRA-1-60 positive colonies selected for calculating reprogramming efficiency.

The wells on day 18 of reprogramming were stained with the anti-TRA-1-60 antibody, and the developed colonies were counted under the microscope. Only the colonies with defined borders, encircled with black ovals (right), were included in counting. Merged colonies were counted as one. TRA-1-60 positive cell clusters without the definitive morphology of an iPSC colony, marked with red X (right), were excluded from calculations. Scale bars, 100 µm.

Supplementary Table 1: iPSC clones generated from parental fibroblasts.

Fibroblast lines	iPSC clones picked	Established iPSC lines (passage 4)	Karyotyped iPSC lines	Lines with satisfactory karyotyping	Karyotyped lines used for pluripotency analyses	Passage at analyses
**FN2**	10	10	5	4	IN2-1	14
					IN2-2	14
					IN2-4	14
					IN2-5	14
**F50**	6	6	3	2	I50-2	15
					I50-3	20
**F50S**	3	2	2	2	I50S-1	14
					I50S-2	19
**FD54**	6	6	4	3	ID54-4	6
					ID54-6	6
**FEH1**	7	6	2	2	IEH1-1	17
					IEH1-7	16
**FEB1**	6	6	3	3	IEB1-2	18
					IEB1-4	16
**FRD1**	5	5	3	3	IRD1-1	18
					IRD1-7	15

Supplementary Table 2: Summary of pluripotency analyses for iPSC lines.

iPSC lines	Pluripotency marker staining	RNA-Seq	mRNA analysis	Teratoma formation	*In vitro* differentiation	Telomere/ Telomerase	STR analysis	Disease causing mutations	Trisomy 21
**IN2-1**	+	+	+	+	+		+		
**IN2-2**	+	+	+	+	+		+		
**IN2-4**	+	+	+	+	+		+		
**IN2-5**	+	+	+	+	+		+		
**I50-2**	+	+	+	+	+	+	+		
**I50-3**	+	+	+	+	+	+	+		
**I50S-1**	+	+	+	+	+	+	+		
**I50S-2**	+	+	+	+	+	+	+		
**ID54-4**	+			+					+
**ID54-6**	+			+					+
**IEH1-1**	+		+	+				+	
**IEH1-7**	+		+	+				+	
**IEB1-2**	+		+	+				+	
**IEB1-4**	+		+	+				+	
**IRD1-1**	+		+	+				+	
**IRD1-7**	+		+	+				+	

Supplementary references

1. Warren, L., Ni, Y., Wang, J. & Guo, X. Feeder-free derivation of human induced pluripotent stem cells with messenger RNA. *Sci Rep* **2**, 657, doi:10.1038/srep00657 (2012).
