# Supplementary material for: High-efficiency RNA-based reprogramming of human primary fibroblasts
Source: Nat Commun. 2018 Feb 21;9:745. doi: 10.1038/s41467-018-03190-3 (PMC5821705; doi:10.1038/s41467-018-03190-3)
Supplement: Supplementary file 3 — Description of Additional Supplementary Files [file 41467_2018_3190_MOESM3_ESM.pdf]

Description of Additional Supplementary Files

File Name: Supplementary Movie 1
Description: Beating cardiomyocyte - like cells derived from IN2-1 iPSCs.

File Name: Supplementary Movie 2
Description: Beating cardiomyocyte - like cells derived from IN2-2 iPSCs.

File Name: Supplementary Movie 3
Description: Beating cardiomyocyte - like cells derived from IN2-4 iPSCs.

File Name: Supplementary Movie 4
Description: Beating cardiomyocyte - like cells derived from I50-2 iPSCs.

File Name: Supplementary Movie 5
Description: Beating cardiomyocyte - like cells derived from I50S-1 iPSCs.

File Name: Supplementary Data 1
Description: Nanostring CodeSet details for gene expression profiling of the generated iPSCs.

File Name: Supplementary Data 2
Description: Nanostring CodeSet details for the time course gene expression experiment.
